# P300- long-latency auditory evoked potential in normal hearing subjects: simultaneous recording value in Fz and Cz

**DOI:** 10.1016/S1808-8694(15)30783-7

**Published:** 2015-10-19

**Authors:** Josilene Luciene Duarte, Kátia de Freitas Alvarenga, Marcos Roberto Banhara, Ana Dolores Passarelli de Melo, Roberta Moreno Sás, Orozimbo Alves Costa Filho

**Affiliations:** 1 MSc. in Speech and Hearing Therapy - Faculdade de Odontologia de Bauru - FOB. Universidade de São Paulo - USP. Speech and Hearing Therapist; 2 Associate Professor - Speech and Hearing Graduate Program - Faculdade de Odontologia de Bauru - FOB / Universidade de São Paulo - USP; 3 MSc. in Experimental Pathophysiology - Faculdade de Medicina FM. Universidade de São Paulo, USP. Speech and Hearing Therapist; 4 MSc. in Speech and Hearing Therapy - Faculdade de Odontologia de Bauru. FOB. Universidade de São Paulo. USP. Speech and Hearing Therapist; 5 Specialist in Audiology - Faculdade de Odontologia de Bauru - FOB. Universidade de São Paulo - USP. Speech and Hearing Therapist; 6 Full Professor - Speech and Hearing Therapy Program - Faculdade de Odontologia de Bauru - FOB. Universidade de São Paulo - USP, MD. Otologist. Coordinator of the Audiologic Reseach Center - CPA. Hospital de Reabilitação de Anomalias Craniofaciais - HRAC - USP. Professor

**Keywords:** cognition, auditory evoked potentials, p300 evoked potential

## Abstract

The P300 is and auditory Evoked Potential, called endogenous potential because it reflects the functional use the individual makes of the auditory stimulus, being highly dependent on cognitive skills; among them we list attention and auditory discrimination. It is a procedure of objective evaluation; however, one that depends on the examiner's experience to detect wave peaks, and it is important to use recording methods that facilitate the response presence analysis and result interpretation.

**Aim:**

to analyze the P300 Long Latency Auditory Evoked Potential obtained through the use of two active electrodes positioned on Fz and Cz.

**Materials and Methods:**

330 individuals from both genders and age ranging between 7 and 34 years participated in this study, they all had normal hearing and did not have any risk factor for mental problems.

**Results:**

Results show that there was no statistically significant difference for N2 and P3 latency and P3 amplitude as far as gender is concerned, nor correlation with the individual's age. There was a strong correlation of these measures with Fz and Cz electrode positioning.

**Conclusion:**

Fz and Cz active electrodes positioning can be considered one more resource to help in the P300 clinical analysis.

## INTRODUCTION

The hearing mechanism is based on the transduction of the acoustic stimulus into neural inputs through the inner ear, the transmission of these inputs through a neural network all the way to the cerebral cortex and the perception registering with later cognitive elaboration of the acoustic signal. Thus, the sound message is perceived and understandable. When talking about hearing skills, we initially think about what happens in the ear, that is, the capacity to detect the sound presence; however, this skill is only part of the processing that happens in the hearing system^1^, ^2^.

Studying Auditory Evoked Potentials allows one to evaluate the entire auditory system, from its periphery as in electrocochleography, all the way to its more central portion, as in the Long Latency Auditory Evoked Potential. The P-300 Long Latency Auditory Evoked Potential is considered a cognitive, endogenous potential, because it reflects the functional use the individual makes of the stimulus, not depending directly on its physical characteristics. For it to be generated, it is necessary to discriminate a rare auditory stimulus, among others which are frequent and of the same modality, with different physical characteristics^3^. In studying the P-300 Long Latency Auditory Evoked Potential, two components can be evaluated, the N2 (or N200), which is associated with the perception, discrimination, recognition and classification of an auditory stimulus; and P3 (or P300) which occurs when the individual consciously recognizes the presence of a change in the auditory stimulus^4^.

It is believed that multiple generators contribute to recording components N2 and P3 belonging to the P-300 Long Latency Auditory Evoked Potential, such as the supratemporal cortex, in the case of component N2, and the reticular formation, lemniscus, inferior colliculus, thalamus, primary cortex, frontal cortex, centro-parietal cortex and hypocampus^4^, ^5^, and that it is associated to information processing and not to the activity of the individual's memory^6^. This potential can be altered when there are deficits in the selective attention and alert mechanisms, state of conscience, and psychological conditions that impair attention^4^, ^5^.

In clinical practice, these potentials are recorded using electrodes which are positioned on the skull surface, according with the International System 10–20 (SI 10–20) of the American Society of Eletroencephalography^7^. Nonetheless, there is no consensus in relation to the number and positioning of the live electrodes, having seen that some authors use only one live electrode placed on Cz^8^, ^9^, ^10^, ^11^, others use two live electrodes, placed on Fz and Cz^12^^,^^3^^,^^13^, Fz and Pz^14^, Cz and Pz^15^^,^^16^, or even three live electrodes, placed on Fz, Cz and Pz^17^.

There are literature reports of great variability in the latency of the P-300 Long Latency Auditory Evoked Potential P3 component, when measured in Fz and in Cz, shown by the high values of the standard deviation which were 33.59 ms and 25.50 ms for Fz and Cz, respectively. The same was observed for the P3 amplitude, especially with the electrode positioned on Fz, which standard deviation value was of 8.16 microvolts^18^.

We must also take into account the age and gender of the individuals when analyzing the P300-Long Latency Auditory Evoked Potential. As far as chronologic age was concerned, most of the studies were held during the 70's and 90's, and showed an increase in latency and amplitude reduction with age^19^, ^20^, ^21^, ^22^, ^23^. In a more recent study^9^ in which children who passed and failed school, with ages varying between eight and thirteen years, we did not observe correlations between the age of the individuals and the P3 component latency. On the other hand, as normal individuals were assessed, with ages varying between eight and eleven years, it was observed an increase in the P3 component latency as age increased; however, not statistically significant^12^.

Nonetheless, the literature studied does not have a consensus in relation to the minimum age for a person to be tested. Some authors^24^ reported that from 15 to 40 years there is an increase in the P3 and N2 components' latencies of 0.8 ms/year a drop of 0.2 μV/year in the N2-P3 complex amplitude. The reverse effect is observed on the ages between 6 and 15 years, where the N2 component latency falls to an average of 18.4 ms/year. Others reported that for ages between 25 and 80 years, there is a latency increase of 1.25 ms per year25, or of 0.9 to 1.8 ms per year26. Nonetheless, other authors^27^^,^^28^ stated that P3 starts to increase only after the second or third decades of life, or it starts at 45 years of age^29^. Some authors stated that the increase in the P-300 Long Latency Auditory Evoked Potential happens in a non-linear fashion with age^24^^,^^22^, and others do not see such linearity^23^.

As far as the individuals' gender was concerned, some papers^21^^,^^30^ did not find statistically significant differences. In a study carried out to measure the P-300 Long Latency Auditory Evoked Potential in a population made up of healthy individuals with ages varying between 21 and 35 years, in which the TDH 39 phone was used, there was no statistically significant difference between the genders when comparing P3 component amplitude and latency, however, such difference does exist when we compare the N2 component^10^. On the other hand, another study showed a statistically significant difference between the genders, and females had lower mean P3 component latency values and standard deviation than males^18^. Contrary to this one, another study did not find these gender differences^12^.

This paper aimed at analyzing the P-300 Long

Latency Auditory Evoked Potential obtained through the use of two active electrodes positioned on Fz and Cz, in normal individuals and check for its true relevance in the clinical analysis of the case.

## MATERIALS AND METHODS

This study was carried out in the Speech and Hearing Therapy clinic of the Bauru Dentistry School - University of São Paulo, and was approved by the Ethics in Human Being Research Committee of the Faculdade de Odontologia de Bauru da Universidade de São Paulo, protocol # 69/2003.

Our series had 33 individuals, 14 males and 19 females, with ages ranging between 7 and 34 years, defined according to the central nervous system maturing process.

All the participants and/or guardians were aware of the procedure and received a patient instruction letter. They all signed the Informed Consent Form.

We used a questionnaire to rule out hearing impairment risk factors or neurologic alterations that could impact the results, and later we performed a conventional audiologic evaluation made up of Threshold Tonal Audiometry, Logoaudiometry and Acoustic Impedance Testing. This assessment was carried out in a sound-treated booth, using the Madsn Audiometer, model Midmate 622, with TDH-39 ear-phones, calibrated in the ANSI-69 standard and the Interacustic, AZ 7 impedance meter. We considered normal hearing threshold to be equal to or below 25 dBHL.

The P 300 Long Latency Auditory Evoked Potential test was carried out in a silent room, with the individual comfortably laying down on a bed, instructed to remain alert, paying attention to the rare stimulus presented in a random fashion to the frequent stimulus (oddball paradigm), and count it out loud.

**Figure 1:**
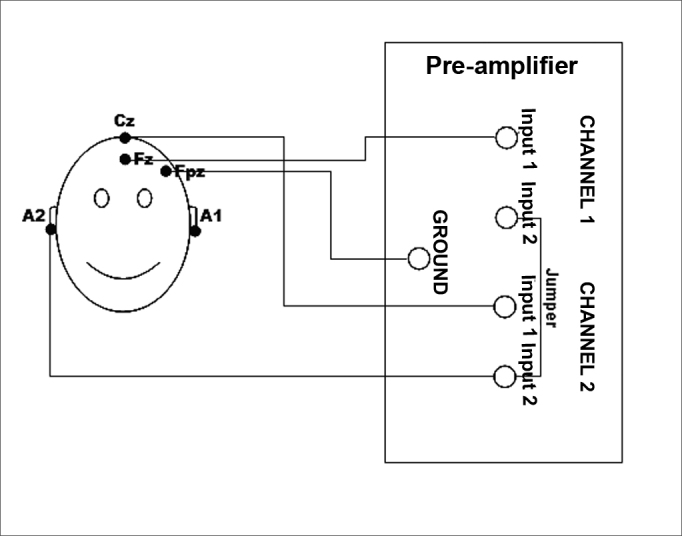
Illustration of the electrodes placement on a patient's skull according to the international 10–20 system and the cable connections on the pre-amplifier cables of the Auditory Evoked Potentials recording system.

In order to study the P300 Long Latency Auditory Evoked Potential, we used the Biologic's Evoked Potential System (EP) device, which test parameters and electrode positioning are described in Chart 1 and Figure 1.

**Chart 1:** Parameters used in the study of the P300 Long Latency Evoked Auditory Potential.

Parameters used in the study of the p300 long latency evoked auditory potential	
Stimulus type	Tone burst (20% raro e 80 % freqüente)
Tone burst (20% rare and 80 % frequent)	70 Dbna
Stimulus frequency	2000 Hz (rare); 1000 hz (frequent)
Stimulus intensity	70 Dbhl
Stimulus occurence speed	1 Stimulus per second
Electrode types	Ekg/agcl with gel
Electrode positioning	Fz and cz (active); a1 and a2 (reference)
Pre-amplifier	Channels 1 and 2 - input 1 active; input 2 reference (jumper)
Impedance	≤ 5 KΩ(individual); ≤ 2 kΩ (between electrodes)
Band pass filter	1 To 25 hz
Transducer	3a insertion phones

**Figure 2:**
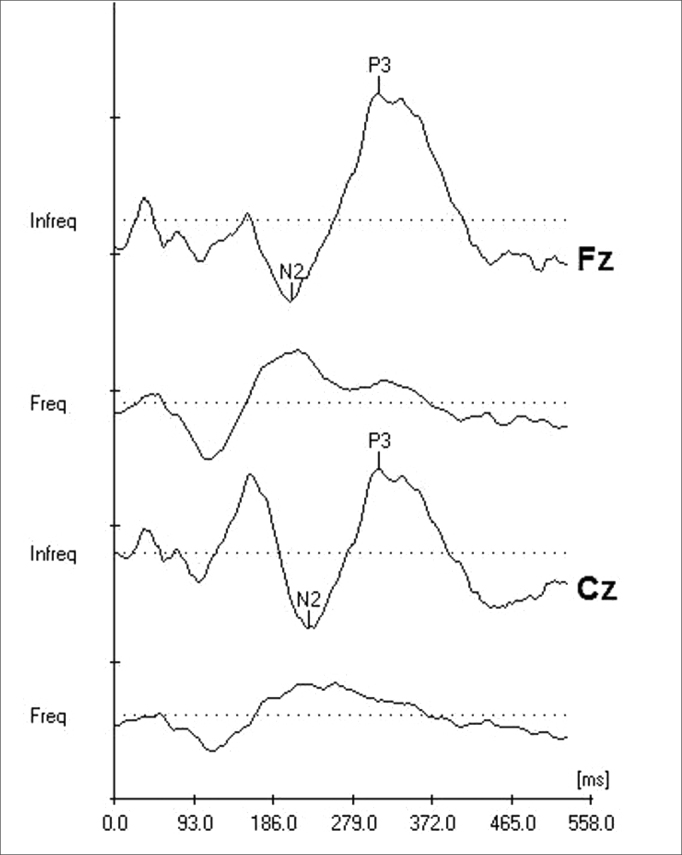
Records of the N2 and P3 components from the P300 Long Latency Evoked Auditory Potential, simultaneously captured by the electrodes positioned on Fz and Cz. Markings of the N2 negative and P3 positive peaks.

As to the analysis parameters, as study objects, we used the absolute latency of P3 and N2 components and P3 amplitude, recorded from Fz and Cz (Figure 2).

We considered the P300 Long Latency Auditory Evoked Potential present when the N2 and P3 components were simultaneously recorded from Fz and Cz. In order to localize the N2-P3 complex in each record, we used the N2 component as the highest negative peak with latency around 200 ms, located before the highest positive peak, P3, with latency around 300ms (Figure 1).

The results were submitted to descriptive statistical analysis (mean, standard deviation, maximum and minimum values); t student tests for gender comparison, at a significance level of p ≤ 0.05; and the Pearson correlation coefficient in order to compare the correlation between age variables and the recordings from Fz and Cz, with significance levels of p ≤ 0.05 and p ≤ 0.01, respectively.

## RESULTS

Table 1 shows the descriptive analyses results (mean, standard deviation, maximum and minimum values) and comparison between males and females by means of the t Student paired test for P3 and N2 component latency (ms)and P3 amplitude (amp-μV). We can see that there was no statistically significant difference as far as gender is concerned, since the p values were higher than 5%.

**Table 1:** Descriptive analysis (mean, standard deviation, maximum and minimum values) and comparison between males and females (paired t student test) for the P3 and N2 component latency and P3 component amplitude (amp).

P300 LONG LATENCY EVOKED AUDITORY POTENTIAL						
Fz		Cz			Cz	
	N2 (ms)	P3 (ms)	P3 amp (μv)	N2 (ms)	P3 (ms)	P3 amp (μv)
Mean ± sd	230±31	339±20	1.81±1.06	228±31	341±23	2,12±1,07
Minimum	183	301	0.1	183	301	0,3
Maximum	275	369	4.3	273	371	3,9
Male x female	0.95	0.92	0.17	0.94	0.69	0,14

P ≤ 0.05 statistically significant

Table 2 shows the Pearson (r) correlation coefficient results when compared to the latency (ms) of N2 and P3 components and P3 amplitude (amp-μV), for the electrodes positioned on Fz and Cz. There was a significant correlation for P3 and N2 component latencies, as well as for P3 amplitude. However, it is possible to see that this correlation was stronger for P3 latency (r = 0.940).

**Table 2:** Pearson's correlation coefficient to compare the N2 and P3 components' latency (ms) and P3 component amplitude (μV), measured in F_z_ and C_z_.

P300 LONG LATENCY EVOKED AUDITORY POTENTIAL			
Electrode positioning	Latency n2	Latêncy p3	Amplitude p3
Fz x cz	0.676*	0.940*	0.687*

* The correlations were statistically significant (P < 0.01).

Table 3 shows the r and p values for the Pearson correlation coefficient considering the age of the individuals, and the N2 and P3 component latencies (ms) and P3 amplitude (amp-μV) P3, measured from Fz and Cz. We did not observe any correlation between age and the components' latency and amplitude.

**Table 3:** Correlation between the individuals' age by means of the Pearson's correlation test, with latency (ms) of components N2 and P3 and P3 component amplitude (amp-μV), measured in F_z_ and C_z_.

P300 LONG LATENCY EVOKED AUDITORY POTENTIAL												
Fz			Cz						Cz			
	N2		P3		P3 amp		N2		P3		P3 amp	
Correlation coefficient	*r*	*p*	*r*	*p*	*r*	*p*	*r*	*p*	*r*	*p*	*r*	*p*
	0.02	0.89	0.29	0.09	-0.19	0.28	0.03	0.86	0.19	0.28	-0,08	0,64

p≤ 0.05 - statistically significant

## DISCUSSION

The P-300 Long Latency Auditory Evoked Potential assesses hearing cognitive processes, providing the clinician with information about the central auditory nervous pathway integrity.

In this study, the values associated with the mean and standard deviation found for the P-300 Long Latency Evoked Auditory Potential from Fz and Cz (Table 1) were of 230 and 31 ms for the N2 and 399 component latency and 20 ms for the P3 latency, respectively. On the other hand, in Cz the values obtained were 228 and 31 ms for the N2 and 341 and 23 ms for the P3 latency, respectively. As is described in the literature18, there was also a large variability for the P-300 Long Latency Evoked Auditory Potential latency, when measured from Fz and Cz.

The results obtained in this study corroborate others^21^^,^^30^^,^^12^, in which the authors did not find statistically significant differences between genders for the P3 and N2 component latencies and P3 amplitude. On the other hand, in other studies^10^^,^^18^ the authors observed a variation in the latency and amplitude of components N2 and/or P3 gender wise.

Considering the age of the individuals evaluated and the P-300 Long Latency Evoked Auditory Potential, for components CzN2, FzN2, CzP3 and FzP3, CzP3amp, FzP3amp, the Pearson Correlation test did not show correlation between age and latency and amplitude values (Table 3), despite the fact that some authors^19^, ^20^, ^21^, ^22^, ^23^, ^24^, ^25^, ^26^^,^^29^ reported that the P3 component can be altered with age in a linear fashion. However, P2 starts to increase only on the second and third decades of life^27^^,^^28^, and this fact can justify the findings in this study, because our population had age varying between seven and 34 years. It is also important to stress that we still need studies that assess a larger number of individuals and a broader age range in order to show the effects of age on the latency of components N2 and P3.

As seen on Table 2, there was a significant correlation with the P3 latency measured from the two recording channels (Fz and Cz).

Although there is no consensus in the literature regarding the number of active electrodes to be used for an effective recording of the P-300 Long Latency Evoked Auditory Potential and their placement on the skull, this study showed, in the series studied, that the use of two active electrodes, in this case Fz and Cz, is a parameter that can be used in clinical practice in order to determine the presence of the P3 component.

The study of P-300 Long Latency Evoked Auditory Potential is an objective procedure, but its analysis is rather subjective, depending on a good clinical experience to visually detect the waves. Thus, this type of analysis can help in obtaining more worthy results in the assessment of the auditory system by means of electrophysiological procedures.

## CONCLUSION

With these results, we can conclude that there was no correlation between P3 and N2 component latencies, as well as P3 amplitude and the age of the individuals; there was no statistically significant difference between genders, and the use of two active electrodes positioned on Fz and Cz, respectively, can be considered one more option to help in the analysis of the P-300 Long Latency Evoked Auditory Potential.
